# Bactericidal Properties of Rod-, Peanut-, and Star-Shaped Gold Nanoparticles Coated with Ceragenin CSA-131 against Multidrug-Resistant Bacterial Strains

**DOI:** 10.3390/pharmaceutics13030425

**Published:** 2021-03-22

**Authors:** Sylwia Joanna Chmielewska, Karol Skłodowski, Joanna Depciuch, Piotr Deptuła, Ewelina Piktel, Krzysztof Fiedoruk, Patrycja Kot, Paulina Paprocka, Kamila Fortunka, Tomasz Wollny, Przemysław Wolak, Magdalena Parlinska-Wojtan, Paul B. Savage, Robert Bucki

**Affiliations:** 1 Department of Medical Microbiology and Nanobiomedical Engineering, Medical University of Bialystok, 15-222 Bialystok, Poland; sylwia.chmielewska@umb.edu.pl (S.J.C.); karol.sklodowsky@gmail.com (K.S.); piotr.deptula@umb.edu.pl (P.D.); ewelina.piktel@wp.pl (E.P.); krzysztof.fiedoruk@umb.edu.pl (K.F.); 2 Institute of Nuclear Physics, Polish Academy of Sciences, 31-342 Krakow, Poland; joannadepciuch@gmail.com (J.D.); magdalena.parlinska@ifj.edu.pl (M.P.-W.); 3 Department of Microbiology and Immunology, Institute of Medical Sciences, Collegium Medicum, Jan Kochanowski University in Kielce, 25-365 Kielce, Poland; patrycja.kot52@gmail.com (P.K.); paulina.paprocka@ujk.edu.pl (P.P.); kamilafortunka@gmail.com (K.F.); przemyslaw.wolak@ujk.edu.pl (P.W.); 4 Holy Cross Cancer Center, Kielce, 25-734 Kielce, Poland; tomwollny@gmail.com; 5 Department of Chemistry and Biochemistry, Brigham Young University, Provo, UT 84602, USA; pbsavage@chem.byu.edu

**Keywords:** nanosystems, ceragenins, CSA-131, MDR (multidrug-resistant), Au NPs

## Abstract

Background: The ever-growing number of infections caused by multidrug-resistant (MDR) bacterial strains requires an increased effort to develop new antibiotics. Herein, we demonstrate that a new class of gold nanoparticles (Au NPs), defined by shape and conjugated with ceragenin CSA-131 (cationic steroid antimicrobial), display strong bactericidal activity against intractable superbugs. Methods: For the purpose of research, we developed nanosystems with rod- (AuR NPs@CSA-131), peanut-(AuP NPs@CSA-131) and star-shaped (AuS NPs@CSA-131) metal cores. Those nanosystems were evaluated against bacterial strains representing various groups of MDR (multidrug-resistant) Gram-positive (MRSA, MRSE, and MLS_b_) and Gram-negative (ESBL, AmpC, and CR) pathogens. Assessment of MICs (minimum inhibitory concentrations)/MBCs (minimum bactericidal concentrations) and killing assays were performed as a measure of their antibacterial activity. In addition to a comprehensive analysis of bacterial responses involving the generation of ROS (reactive oxygen species), plasma membrane permeabilization and depolarization, as well as the release of protein content, were performed to investigate the molecular mechanisms of action of the nanosystems. Finally, their hemocompatibility was assessed by a hemolysis assay. Results: All of the tested nanosystems exerted potent bactericidal activity in a manner resulting in the generation of ROS, followed by damage of the bacterial membranes and the leakage of intracellular content. Notably, the killing action occurred with all of the bacterial strains evaluated, including those known to be drug resistant, and at concentrations that did not impact the growth of host cells. Conclusions: Conjugation of CSA-131 with Au NPs by covalent bond between the COOH group from MHDA and NH_3_ from CSA-131 potentiates the antimicrobial activity of this ceragenin if compared to its action alone. Results validate the development of AuR NPs@CSA-131, AuP NPs@CSA-131, and AuS NPs@CSA-131 as potential novel nanoantibiotics that might effectively eradicate MDR bacteria.

## 1. Introduction

The menace of MDR (multidrug-resistant) bacteria in therapy failures leading to patient mortality cannot be underestimated [1,2]. The Interagency Coordination Group (IACG) on antimicrobial resistance warns and highlights with great severity that drug-resistant diseases could trigger 10 million deaths each year by 2050 [3]. Moreover, recent predictions indicate that deaths on account of bacterial infections will be more numerous in comparison to the mortality due to cancer by 2050 [4]. Nowadays, more and more common diseases, including urinary or respiratory tract infections, are untreatable and pose a significant burden. The fact that, worldwide, at least 700,000 people (in the U.S., more than 35,000) die each year due to drug-resistant illnesses reflects the depth and scope of this issue [3,5]. One of the most threatening types of infection are those induced by methicillin-resistant *Staphylococcus aureus* (MRSA) or *Staphylococcus epidermidis* (MRSE), characterized by their resistance to virtually all β-lactam antibiotics [6]. Although MRSA infections overall have decreased over the past few years, advances in the prevention of MRSA bloodstream infections are still insufficient [7,8]. Significantly, *S. aureus* bacteremia (SAB) frequently leads to metastatic infections, along with endocarditis, septic arthritis, and osteomyelitis. Moreover, SAB can be responsible for hazardous complications, including sepsis and septic shock [7]. Nowadays, the successful treatment of SAB infections, especially those caused by MRSA, remains an unsolved challenge. MRSA is known as one of the most frequent MDR bacteria isolated in acute care hospitals, long-term care hospitals, and rehabilitation institutions [4,9]. It is estimated that in hospitals in the USA, approximately 50% of *S. aureus* strains are methicillin-resistant, and nearly 20,000 people died due to bloodstream infections caused by this pathogen in 2017 [4]. In Europe, the proportion of MRSA isolates has dropped over time; however, 7 of the 30 European Union countries still report 25% or more of invasive *S. aureus* isolates as MRSA [7,10].

Although alternatives to β-lactam antibiotics such as macrolides, lincosamides, or streptogramins group B (MLS_b_) can be used to treat diseases caused by *Staphylococcus* spp., the past few decades have witnessed the widespread emergence of resistance to these and other antibiotics, which makes infections tough to treat and control [11]. Another common cause of healthcare-associated infections, also characterized by a reduced susceptibility to β-lactam antibiotics, are Gram-negative bacteria producing various types of β-lactamases, such as extended-spectrum β-lactamases (ESBLs) or AmpC cephalosporinases [9]. It should be emphasized that any Gram-negative organism has the potential to harbor ESBL genes; nevertheless, this mechanism of resistance is most prevalent in *Escherichia coli*, *Klebsiella pneumoniae*, *Klebsiella oxytoca*, and *Proteus mirabilis* [12]. The strains harboring ESBLs are reported as resistant to all penicillins, cephalosporins (except cephamycin), and aztreonam. Although most ESBLs are inhibited by β-lactamase inhibitors (clavulanic acid, sulbactam, and tazobactam), their combinations with penicillins are not recommended for treating serious infections caused by ESBL (+) strains [13]. Until the year 2000, ESBL-producing *Enterobacteriaceae* were mainly responsible for nosocomial infections. However, ESBL (+) bacteria spread quickly to the community, hindering the effective therapy of outpatients [14]. In the USA, the occurrence of ESBL infections increased by 53% from 2012 to 2017 [12]. Additionally, approximately 14% of healthy individuals are colonized by strains harboring ESBLs [9].

Similarly, the emergence and dissemination of MDR and extensively drug-resistant (XDR) *Pseudomonas aeruginosa*strains have recently become a menace and concern for public health [15,16]. In 2018, the European Center for Disease Prevention and Control (ECDC) stated that ≥25% of *P. aeruginosa* isolates were resistant to at least three antimicrobial groups among 6 of the 30 European Union countries (including Poland) [17]. *P. aeruginosa* has a remarkable arsenal of mechanisms of antibiotic resistance; for example, an overproduction of chromosomal AmpC cephalosporinase is presumably the most widespread mutation-driven β-lactam resistance mechanism, reported in over 20% of *P.* *aeruginosa* clinical isolates [15]. Furthermore, the World Health Organization (WHO) defined carbapenem-resistant *P. aeruginosa *as a pathogen of critical priority that urgently requires new treatment options. The lack of efficient therapies against MDR *P.* *aeruginosa* infections leads to worse outcomes and higher mortality and morbidity rates [18]. The resistance to carbapenems, e.g., imipenem or meropenem, can be associated with the loss (or mutations) of the carbapenem-specific porin OprD (outer membrane porin D). For instance, the prevalence of imipenem resistance, due to OprD deficiency, among *P.* *aeruginosa* is estimated at above 20%. Moreover, OprD inactivation, commonly accompanied by AmpC overexpression, can make *P.* *aeruginosa* resistant to all β-lactams [15]. 

The ongoing emergence of drug-resistant bacteria poses a considerable threat to healthcare and the global economy [18]. Regrettably, the pace of antibiotic development has not followed the emergence of bacterial resistance [19]. Therefore, the development of novel antimicrobials to alleviate the spread of antibiotic resistance has become a global priority [18]. Gold nanoparticles (Au NPs) are promising candidates for addressing this crisis [20]. In general, a vast range of metal NPs have been synthesized, e.g., gold (Au), silver (Ag), zinc (Zn), copper (Cu), magnesium (Mg), titanium (Ti), or bismuth (Bi) [4,21]. Nonetheless, Au NPs have been highlighted as the best potential candidate for treating infectious diseases in view of the following properties. Au NPs have gained increased attention due to their ultra-small size that results in novel properties, such as greater penetration into cells, high surface-to-volume ratios, and an enhanced number of active atoms on their outer surfaces [22,23]. Furthermore, nanoparticles have a specific impact on growing bacteria by affecting the integrity of the cell membrane and thereby resulting in DNA damage. Moreover, the non-toxic nature of Au NPs makes them an outstanding candidate for combating MDR bacteria [24]. It must be emphasized that Au NPs have a lower toxicity compared to other metallic nanomaterials, for instance, silver nanoparticles (Ag NPs) [22]. The deposition of Ag NPs in the liver, spleen, and lungs, ultimately leading to organ damage and dysfunction, significantly reduces their therapeutic applications [25]. The therapeutic potential of Au NPs is also highlighted by their stability and biocompatibility, as well as ease of surface modification [26]. Therefore, Au NPs are broadly used in many biological applications, particularly in medical and gene therapy [22]. Importantly, Au NPs may be applied as biosensors or vessels for drug delivery and even selective treatment of cancer [27,28,29]. According to the literature, Au NPs have been reported as effectual antibacterial agents against antibiotic-resistant strains, such as *S. aureus, Enterococcus faecium, Enterococcus faecalis, Escherichia coli, Salmonella typhimurium, Shigella dysenteriae*, *Vibrio cholerae* and *Mycobacterium tuberculosis* [21,30]. It should be pointed out that Au NPs have been hallmarked as a very attractive alternative in developing new methods of infectious disease treatment [27,30,31].

It is widely assumed that NPs can kill bacteria; nevertheless, the fundamental mechanism of antimicrobial action remains obscure, particularly for large NPs that, in some cases, have a limited ability to translocate across the bacterial cell membrane. Interestingly, the mechanical killing of bacterial cells (*S. aureus*, *P. aeruginosa*) by non-translocated NPs, i.e., quasi-spherical and star-shaped gold (Au) NPs was elucidated by Linklater et al. using a combination of complementary experimental and theoretical investigations. Overall, the proposed mechanism involves mechanical deformation caused by an increase in membrane tension triggered by the adsorption of NPs which results in the membrane stretching or squeezing, and finally rupture and cell death [32]. 

The functionalization of Au NPs with antimicrobial ceragenins (CSA’s) is considered a promising approach for amplifying antimicrobial effects [25,26]. In line with this research direction, the current study was aimed at investigating the antibacterial activity of Au NPs functionalized with ceragenins, also referred as cationic steroid antimicrobials. The ceragenins were designed as non-peptide mimics of antimicrobial peptides and display broad-spectrum activity against Gram-positive and Gram-negative bacteria, including MDR strains, as well as fungi, parasites, and lipid-enveloped viruses. Moreover, CSAs also have sporicidal and antibiofilm potential [33]. A key mechanism of CSA’s action is microbial membrane insertion, leading to membrane depolarization [34]. At the same time, CSAs show low toxicity, which supports the clinical application of these compounds [33]. Multiple generations of CSAs have been synthesized and evaluated. The compounds between CSA-1 and CSA-50 have been classified as members of the first generation of CSAs. Whereas the remaining members belong to the second generation of CSAs [34]. A variety of studies confirmed that the second-generation of CSAs, notably CSA-131, possess potent antimicrobial activity against a broad spectrum of microorganisms such as carbapenem-resistant *E. coli, K. pneumoniae, E. cloacae* [35], or *P.* *aeruginosa* [36]; colistin-resistant *K. pneumoniae* and *Acinetobacter baumannii* [37,38]; as well as uropathogenic *E. coli* strains [39]. Moreover, Hacioglu et al. have demonstrated the antibiofilm activity of CSA-131 against *C. albicans* cells in both mono- and multispecies biofilms, i.e., comprising of *C. albicans* and MRSA or MSSA (methicillin-susceptible *S. aureus*) strains [34]. Additionally, Hashemi et al. argued that CSA-131, formulated in micelles, may be beneficial in combating polymicrobial and biofilm-related infections in the lung and trachea, notably those associated with cystic fibrosis (CF). Their therapeutic potential is also further highlighted by the fact that CSA-131 formulating in poloxamer micelles does not impact on cilia, whilst preserving the capacity to substantially reduce the numbers of pathogenic fungal strains. Significantly, a crucial criterion in the effective treatment of respiratory lung infections is the use of antimicrobial substances that reliably eradicate biofilms without interfering with cilia [40]. Likewise, findings observed by Oyardi et al. emphasized that CSA-131 and CSA-131 in poloxamer form may act as a potential agent in the treatment of *Stenotrophomonas maltophilia* infections due to its low cytotoxicity on the CF cell line and significant antimicrobial or antibiofilm properties [41]. With respect to fungi strains, apart from *C. albicans,* CSA-131 also displays notable activity against *Candida parapsilosis*, *Candida tropicalis*, *Candida glabrata*, *Candida krusei,* and *Candida dubliensis* [33], or even the emerging pathogen *Candida auris* [40]. Here, CSA-131 was selected for further research due to its unique properties and the convincing number of the abovementioned reports that justify the possibility of developing new treatments that can be used in infections caused by antibiotic-resistant microorganisms.

It should be highlighted that our previous studies revealed noticeable antibacterial effects of CSA-131 against MDR strains [35]. However, it has not been studied whether Au NPs, functionalized by CSA-131, have potent antimicrobial activity suitable for more efficient treatment of infections. Thus, we investigated the antibacterial features of Au NPs@CSA-131 in three different shapes: rods (AuR NPs@CSA-131), peanuts (AuP NPs@CSA-131), and stars (AuS NPs@CSA-131) against six bacterial strains characterized by various mechanisms of resistance to antibiotics. The motivation for choosing these shapes of nanoparticles was dictated by the previous studies. To date, it is well documented that the shape of the Au NPs can substantially impact their therapeutic efficiency and biocompatibility. Studies performed by Penders et al. [27], which compared the antimicrobial activity of Au NPs in various shapes (including spheres, stars, and flowers), demonstrated that spherical Au NPs did not elicit any effects toward *S. aureus,* whereas flowers and stars were characterized by pronounced antibacterial potency [27]. Other published research also confirmed a decreased cytotoxicity for rods over spheres, which simultaneously increased uptake into cells [42]. It is important to note that for the smaller size of NPs, notably those <5 nm, an increased cytotoxicity has been observed [27]. In the view of the above, we have decided to evaluate the bactericidal activity of nanosystems containing ceragenin CSA-131 attached to the surface of non-spherical gold nanoparticles, which varied not only in shape (rods, peanuts and stars), but also by different sizes of 37–53 nm, 55–65 nm, and 243 nm for AuR NPs, AuP NPs, and AuS NPs, respectively. We believe that the comparison of such prepared nanosystems might help us to understand how variably shaped gold nanoparticles might govern the bactericidal activity of membrane-active compounds and, as such, be employed as effective drug nanocarriers. Considering the previous reports indicating the overcoming of drug resistance using gold nanoparticle-bound antibiotics [43], we aim to explore whether the developed gold-based nanosystems will be effective against multidrug-resistant pathogens.

## 2. Materials and Methods 

### 2.1. Bacterial Strains, Media, and Growth Conditions

Six reference and clinical bacterial isolates, including *S. aureus* Xen 30 (a reference MRSA strain, MLS_b_-positive), *S. epidermidis* 175 (a clinical MRSE, MLS_b_-positive), *K. pneumoniae* ATCC 700603 (a reference ESBL-positive strain, resistant to cephamycins), *K. oxytoca* 329 (a clinical ESBL- and AmpC-positive isolate), *P. aeruginosa* LESB58 (an epidemic cystic fibrosis strain, highly resistant to antibiotics with a high level of AmpC-β-lactamase), and *P. aeruginosa* 510 (a clinical isolate ESBL-positive and resistant to carbapenems) were used. Detailed characteristics of the strains are given in Table 1. The identification and susceptibility patterns of the clinical isolates were performed with a Vitek^®^ 2 Compact automated system (bioMérieux, Marcy-l’Etoile, France)—data are presented in Table 2, Table 3 and Table 4 in accordance with the European Committee on Antimicrobial Susceptibility Testing (EUCAST). The strains of *Staphyloccus* spp., *Klebsiella* spp., and *P. aeruginosa* were cultured and maintained on the recommended selective media, i.e., Chapman, MacConkey, and Cetrimide agar (Biomaxima, Lublin, Poland), respectively. These agar media were also used to culture bacteria (an overnight incubation at 37 °C, unless otherwise indicated) in experiments requiring the quantification of bacterial growth, e.g., killing assay (see below).

### 2.2. Antibacterial Compounds

#### 2.2.1. Ceragenin (CSA-131)

Ceragenin CSA-131 was synthesized as described previously [15] and dissolved in phosphate-buffered saline (Thermo Fisher Scientific, Waltham, MA, USA) using a washer IS-4 sonicator (InterSonic, Olsztyn, Polska) for 30 min at room temperature.

#### 2.2.2. Gold Nanoparticles (Au NPs) Functionalized by CSA-131

The syntheses of rod-, peanut-, and star-shaped gold nanoparticles were achieved using seed-mediated, two-step methods. In the first step, spherical gold nanoseeds were generated as follows: 0.2 M solution of cetrimonium bromide (CTAB) was mixed with 5 mL of 0.5 mM HAuCl_4_ solution and 0.6 mL of 0.1 M NaBH_4_ solution. The reaction was conducted under vigorous stirring and stopped when the solution changed color to red. In the second step, 5 mL of 0.2 M CTAB in water was mixed with 0.2 mL of 0.04 M AgNO_3_, 5 mL of 1 mM HAuCl_4_, 70 µL of 78 mM ascorbic acid (C_6_H_8_O_6_), and 30 µL of Au nanoseeds, and the reaction was allowed to proceed for 30 min (rod-shaped nanoparticles‑AuR NPs) or 3 h (peanut-shaped nanoparticles‑AuP NPs). For star-shaped nanoparticles (AuS NPs) the reaction was stopped after 30 min; however, not 70 µL, but 210 µL, of 78 mM C_6_H_8_O_6_ was added. After this, all nanoparticles were functionalized using 16-mercaptohexadecanoic acid (MHDA) through overnight incubation at 4 °C. MHDA was dissolved in water and the efficiency of functionalization was confirmed by Raman spectroscopy. In the subsequent step, the MHDA-covered Au NPs were rinsed with dimethylformamide (DMF) and incubated in DMF solution of pentafluorophenyl (PFP), *N,N-*diisopropylethylamine (DIPEA) and *N*-cyclohexyl-*N*′-(2-morpholinoethyl) carbodiimide methyl-p-toluenesulfonate (CMC) for 30 min at 25 °C to activate the Au NPs-MHDA complex to attach to the ceragenin. After repeated rinsing with DMF and centrifugation, CSA-131 solution was added and incubated for 30 min at 25 °C. 

In effect, the nanosystems prepared by us consist of two components: CSA-131 (concentration: 2 mg/mL, which is 0.002M, i.e., 12.04 × 10^20^ molecules) and non-spherical nanoparticles (2.93 ng/mL, which is 0.015 × 10^−6^ M, which is 0.0903 × 10^17^ molecules). As a result, we obtained loading of CSA-131 on the surface of the nanoparticles at 133.33 × 10^3^ molecules of CSA-131 per 1 nanoparticle. During the preparation of the nanosystems, we used MHDA in excess to assure that all CSA-131 molecules would attach to the surface of the nanoparticles. Therefore, the amount of ceragenin in the prepared nanosystem was not further calculated and was determined by the amount of ceragenin that was used for the synthesis.

#### 2.2.3. Physicochemical Properties of AuR NPs@CSA-131, AuP NPs@CSA-131 and AuS NPs@CSA-131 Nanoparticles

**Figure 1:**
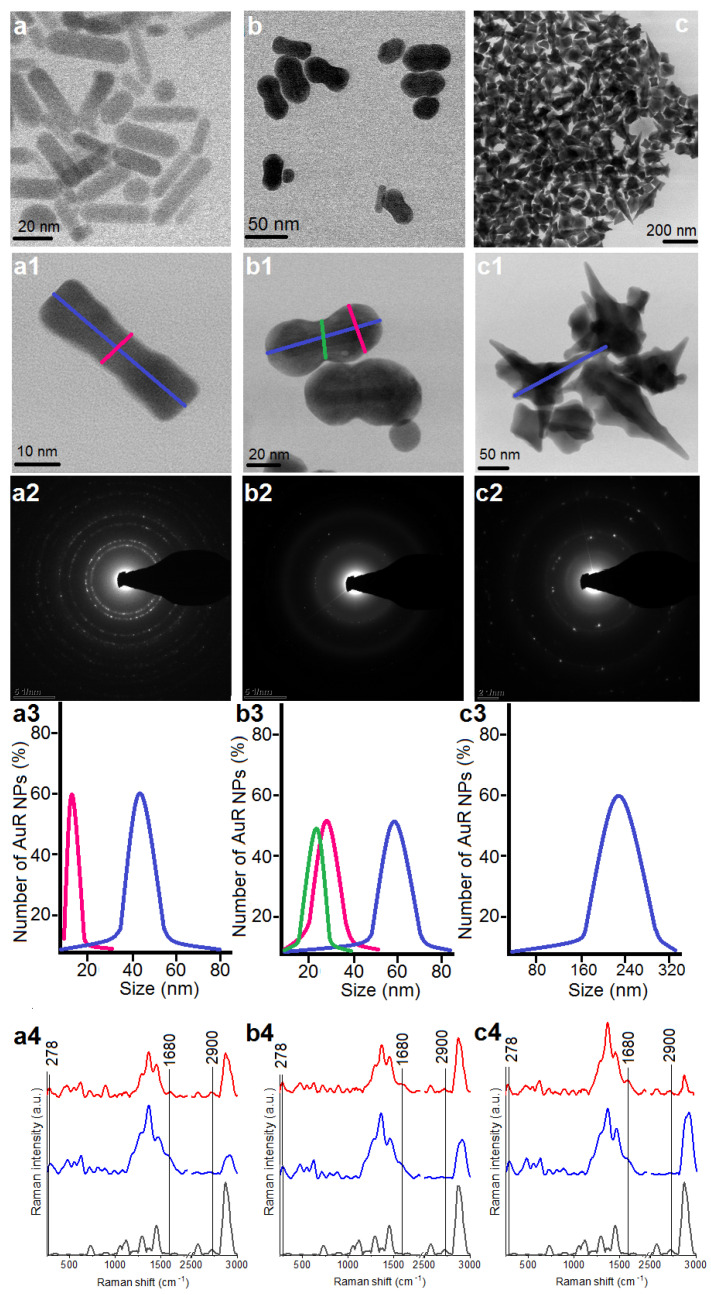
Overview STEM image of the obtained AuR NPs (**a**,**a1**), AuP NPs (**b**,**b1**), and AuS NPs (**c**,**c1**); Selected area diffraction (SEAD) patterns of the AuR NPs (**a2**), AuP NPs (**b2**), AuS NPs (**c2**); size distribution of the AuR NPs (**a3**), AuP NPs (**b3**), AuS NPs (**c3**); and unenhanced Raman spectra of the MHDA (black spectra)-functionalized nanoparticles by MHDA (blue spectra) and immobilized CSA-131 in the NPs surface (red spectra) for the AuR NPs (**a4**), AuP NPs (**b4**), and AuS NPs (**c4**).

The morphology of the differently shaped particles was examined by scanning transmission electron microscopy (STEM) using a high-angle annular dark-field detector (HAADF) in the conventional mode. The measurements were performed on an aberration-corrected FEI Titan electron microscope operating at 300 kV equipped with an field emission gun (FEG) cathode (FEI, Hillsboro, OR, USA). Selected area electron diffraction (SAED) patterns were taken in the transmission electron microscopy (TEM) mode to determine the crystal structure of the Au NPs. The particle size distribution was evaluated based on STEM images taken from different areas of the TEM grids. The Au NPs were measured and analyzed using TIA Software. The diameter of about 100 Au NPs was examined as marked in Figure 1(a1,c1). Fourier transform Raman (FT-Raman) spectra were used to determine the extent of and stability of nanoparticle functionalization and the CSA-131 immobilization processes. Spectra were recorded using a Nicolet NXR 9650 FT-Raman spectrometer equipped with an Nd:YAG laser (1064 nm) and a germanium detector. The measurements were performed in the range of 150 to 3700 cm^−1^ with a laser power of 1 W. An unfocused laser beam was used with a diameter of approximately 100 µm and a spectral resolution of 8 cm^−1^. Raman spectra were processed by Omnic/Thermo Scientific software based on 128 scans. In each obtained spectrum, baseline correction using rubber band methods, as well as vector normalization, were performed through OPUS software.

### 2.3. Estimation of Antibacterial Activity of the Nanosystems

#### 2.3.1. Antimicrobial Susceptibility Testing

The antibacterial efficiency of these nanosystems, i.e., AuR NPs@CSA-131, AuP NPs@CSA-131, and AuS NPs@CSA-131, as well as CSA-131 alone, against all tested bacterial strains (at the final inoculum 5 × 10^5^ CFU/mL) was determined by the estimation of the minimal inhibitory concentrations (MICs) and minimal bactericidal concentrations (MBCs) using a serial microdilution method in Mueller–Hinton broth (Sigma-Aldrich, Saint Louis, MO, USA) on 96-well microtiter plates with final volumes of 200 µL. MIC values, i.e., the lowest concentration of compounds inhibiting the development of visible growth, were recorded after an overnight incubation at 37 °C. MBC values, defined as the lowest concentration of the compounds studied resulting in at least a 99.9% killing of the initial bacterial inoculum, were assessed by plating 10 µL of the dilutions without visible growth in the MIC test on the appropriate selective agar media.

#### 2.3.2. Killing Assay

The MIC/MBC assays were supported by monitoring the bactericidal activity of the agents by colony counting method (killing assay). Briefly, individual colonies of the bacteria at mid-log phase were grown to ~10^8^ CFU/mL, serially diluted to 10^5^ CFU/mL, and exposed to CSA-131 alone, and gold nanoparticles functionalized by CSA-131 at concentrations ranging from 1 to 10 µg/mL. After 60 min of incubation at 37 °C, the plates were transferred to ice and the samples were diluted from 10- to 1000-fold. Thereafter, 10 µL aliquots of each bacterial dilution were plated on the dedicated agar media. The survival rate of the bacteria was calculated by counting the colony-forming units (CFU) as follows: CFU × dilution factor (the inverse of the dilution) = CFU/mL of the sample before exposure.

### 2.4. Analysis of the Bacterial Response to Tested Nanosystems at Molecular Level

The impact of the NP and CSA-131 on bacterial cells was analyzed by the measurement of (i) oxidative stress, through ROS generation assessment, (ii) integrity of the cell membranes, by means of outer and/or plasma membrane permeabilization and depolarization assays, as well as (iii) protein leakage using Bradford’s reagent. Briefly, in the Bradford assay we detect the proteins that were translocated from the cytosole to the extracellular environment and might be detected in the collected supernatant. The mechanism of paramount importance is the binding of coomassie brilliant blue G-250 dye with arginyl and lysyl residues of the proteins (not to the free amino acids) by electrostatic interactions. Moreover, to a lesser extent the hydrophobic interactions of the dye with tryptophan, phenylalanine, and tyrosine residues may be noted [44,45].

All experiments were performed in 96-well microtiter plates with a final volume of 100 µL using bacterial suspensions in PBS and exposed to the concentrations of CSA-131 alone and its complexes with rod-, peanut-, and star-shaped Au NPs ranging from 1 to 10 µg/mL. Absorbance and fluorescence measurements were recorded with a Varioskan Lux microplate reader (Thermo Fisher Scientific, Waltham, MA, USA). Table 5 provides a detailed description of the used methods.

### 2.5. Haemolytic Activity of CSA-131 Nanosystems

A whole blood hemolysis assay was used as an indicator of toxicity of AuR NPs@CSA-131, AuP NPs@CSA-131, AuS NPs@CSA-131, and CSA-131 against the host cells. Hemolytic activity of the studied nanosystems was determined using human red blood cells (RBCs) isolated from the blood of three healthy volunteers. The analyzed compounds in the concentration of 1–50 µg/mL were incubated with RBCs (suspended in PBS, hematocrit ~5%) for 1, 6, 12, and 24 h at 37 °C. To evaluate the release of hemoglobin from the damaged RBCs, the optical absorbance of supernatants (centrifugation at 2500 rpm, 10 min) was measured at 540 nm (Labsystem Varioscan Lux). Simultaneously, a positive control (100% of haemolysis) was taken from the wells in which 1% Triton X-100 (Sigma-Aldrich, USA) was used to disrupt the cell membrane, whereas the RBCs in PBS were used as a negative control. The relative absorbance compared to that treated with 1% Triton X-100 was defined as the percentage of hemolysis.

### 2.6. Statistical Analysis 

All statistical analyses were conducted using Graph Pad Prism, version 8 (GraphPad Software, Inc., San Diego, CA). The data collected were reported as the mean ± standard deviation (SD) of three to six experiments. The significance of differences was determined using the two-tailed Student’s test and a *p*-value **≤** 0.05 was considered to be statistically significant.

## 3. Results

### 3.1. Physicochemical Nature of Rod-, Peanut-, and Star-Shaped Au NPs

The morphology of the synthesized Au NPs was analyzed by STEM using a bright field (BF) detector, Figure 1(a1,b1,c1). Obtained nanoparticles were characterized as rod-shaped, peanut-shaped, and star-shaped, as indicated in Figure 1, panels a1, b1, and c1, respectively. However, to be more precise, the shape of 80% of the rod-shaped nanoparticles and approximately 60% of the peanut-shaped nanoparticles satisfied this description. Additionally, between AuR NPs, a rod-shape with rounded ends was observed as well. In turn, in relation to the AuP NPs, the spherical Au NPs were also noticed. In the second step of our synthesis, the aforementioned spherical Au NPs were used as nanoseeds. Furthermore, the BF STEM images of AuS NPs (Figure 1, panel c1) point out that the gold nanostars are faceted particles. As expected, the crystalline structure (Figure 1, panels a2–c2) and the size of the gold nanoparticles (Figure 1, panels a3–c3) were linked to their shape. For example, the rod-shaped nanoparticles (Figure 1, panel a1) had a crystalline structure and the circles indexed with planes corresponding to the face-centered cubic (fcc) structure of Au were visible in their SAED patterns (Figure 1, panel a2) [46]. On the other hand, although the peanut-shaped nanoparticles (Figure 1, panel b2) also had a crystalline structure, the circles were less sharp in spite of the bigger size. This might be explained by the fact that the AuP NPs were thicker, in particular at their ends. Finally, the SAED pattern of AuS NPs were characterized by sharp rings with visible points (Figure 1, panel b2). This means that the gold nanostars had a crystalline structure and relatively large sizes (Figure 1(c2)). Overall, the size, measured on the longitudinal and transverse axes, of the Au NPs ranged from 45 ± 8 nm and 10 ± 3, respectively, in the case of the AuR NPs (Figure 1, panel a3), through 55–65 nm and 24–34 nm for the AuP NPs (Figure 1, panel b3), to around 243 nm for the AuS NPs (Figure 1, panel c3).

The nanoparticles with different shapes and sizes were functionalized using MHDA and immobilized with ceragenin by covalent bonding between the COOH group from MHDA and NH_3_ from CSA-131. FT‑Raman measurements were performed to verify the successful functionalization and immobilization processes (Figure 1a4–c4). In the unenhanced Raman spectrum of the MHDA sample, a peak of the thiol group (–SH) at 2900 cm^−1^ was visible (black spectra). The disappearance of this peak in the samples of AuR NPs, AuP NPs, and AuS NPs biofunctionalized by MHDA, confirmed an efficient attachment of the MHDA to the surface of the gold nanoparticles (blue spectra presented in Figure 1). Furthermore, in the FT-Raman blue spectra, peaks at 278 cm^−1^ corresponding to Au-S stretching vibrations were visible. These bonds are responsible for creating a connection between the surface of the gold nanoparticles and sulfur from the MHDA [47]. Moreover, in the surface enhanced Raman spectra (SERS) of the AuR NPs@CSA-131, AuP NPs@CSA-131, and AuS NPs@CSA-131 (red spectrum), peaks corresponding to the N-H vibrations (1680 cm^−1^) were observed [48]. These groups were responsible for linking CSA-131 with the biofunctional surfactants on the surface of the AuR NPs, AuP NPs, and AuS NPs [49]. Both observations provide evidence of successful nanoparticle biofunctionalization and CSA-131 immobilization.

### 3.2. Antimicrobial Activity of the Rod-, Peanut-, and Star- Shaped Au NPs 

As presented in Table 6, all Au NP@CSA-131 nanosystems were highly effective against bacterial isolates, and the MIC values ranged from 0.2 µg/mL to 0.4 µg/mL in the case of *Staphycoloccus* spp., 0.4–0.8 µg/mL for *Klebsiella* spp., and 0.8–1.6 µg/mL with regard to *Pseudomonas* spp. In addition, approximately 2.5–3.2-fold augmentation of the bactericidal potency of CSA-131 by Au NPs, in particular by AuR NP@CSA-131 nanosystems, in the MBC assays was observed (Table 6). 

**Figure 2:**
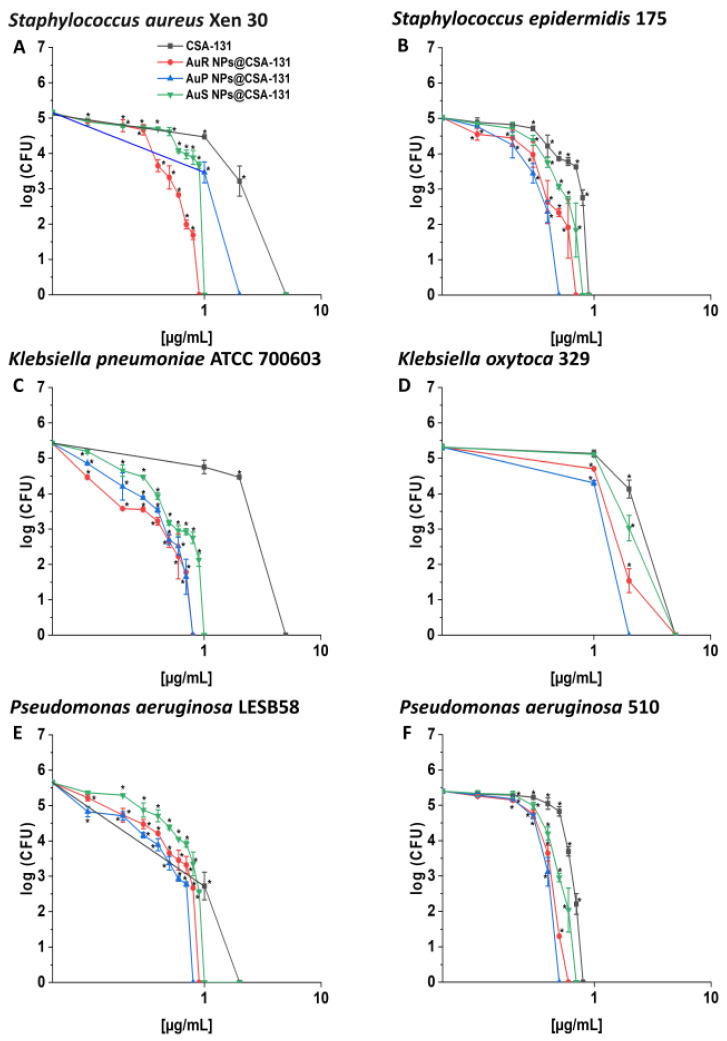
Bactericidal activity of AuR NP@CSA-131, AuP NP@CSA-131, AuS NP@CSA-131, and CSA-131 against *Staphylococcus aureus* Xen 30 (**A**), *Staphylococcus epidermidis* 175 (**B**), *Klebsiella pneumoniae* ATCC 700603 (**C**), *Klebsiella oxytoca* 329 (**D**), *Pseudomonas aeruginosa* LESB58 (**E**), and *Pseudomonas aeruginosa* 510 (**F**). Bactericidal activity of the nanosystems at concentrations of 0.1–10 µg/mL was determined using a standard colony counting assay. Results show the mean ± SD from six measurements. * indicates statistical significance at *p* ≤ 0.05.

AuP NP@CSA-131 was recognized as the most bactericidal against nearly all tested strains in the killing assay (Figure 2, panels A–F), showing 1.5–6.25-fold higher activity in comparison to CSA-131 alone, which was insufficient to eliminate most strains tested at a concentration of 1 µg/mL. This effect was especially visible in the survival rate of *P. aeruginosa* 510 and *K. pneumoniae* ATCC 700603; after treatment with AuP NP@CSA-131 and CSA-131 alone at 0.5 µg/mL, complete eradication or only 0.19% of the bacteria remained with the NP, versus 29.36% and 59.41% of the bacteria surviving with the ceragenin alone, respectively.

To investigate whether the increased efficiency of CSA-containing nanosystems resulted from the antimicrobial activity of the gold nanoparticles themselves, additional killing assay analysis was performed, where tested MDR microorganisms were exposed to CTAB-functionalized nanoparticles (Au NPs + CTAB) or MHDA-modified nanoparticles (Au NPs + MHDA) without further CSA-131 attachment. At gold concentrations corresponding to nanosystem doses, any bactericidal effect was recorded against any tested pathogens (data not shown). 

### 3.3. Bactericidal Mechanism of the Nanosystems Involves the Induction of Oxidative Stress as well as the Destruction and Depolarization of Bacterial Membranes and Protein Leakage 

To understand the mechanism of AuR NP@CSA-131, AuP NP@CSA-131, and AuS NP@CSA-131 antibacterial activity, the following studies were performed: ROS generation assessment, NPN (1-N-phenylnaphthylamine) uptake assay, diSC_(3)_ (3,3-dipropylthiadicarbocyanine iodide) assay, SYTO9/PI (propionium iodine)-dual staining, and a protein leakage assay. In general, in all experiments, the action of rod-, peanut-, and star-shaped Au NPs functionalized by CSA-131 was significantly higher than CSA-131 in a free form. However, certain shape-related differences in the activity of the Au NPs were noted. 

**Figure 3:**
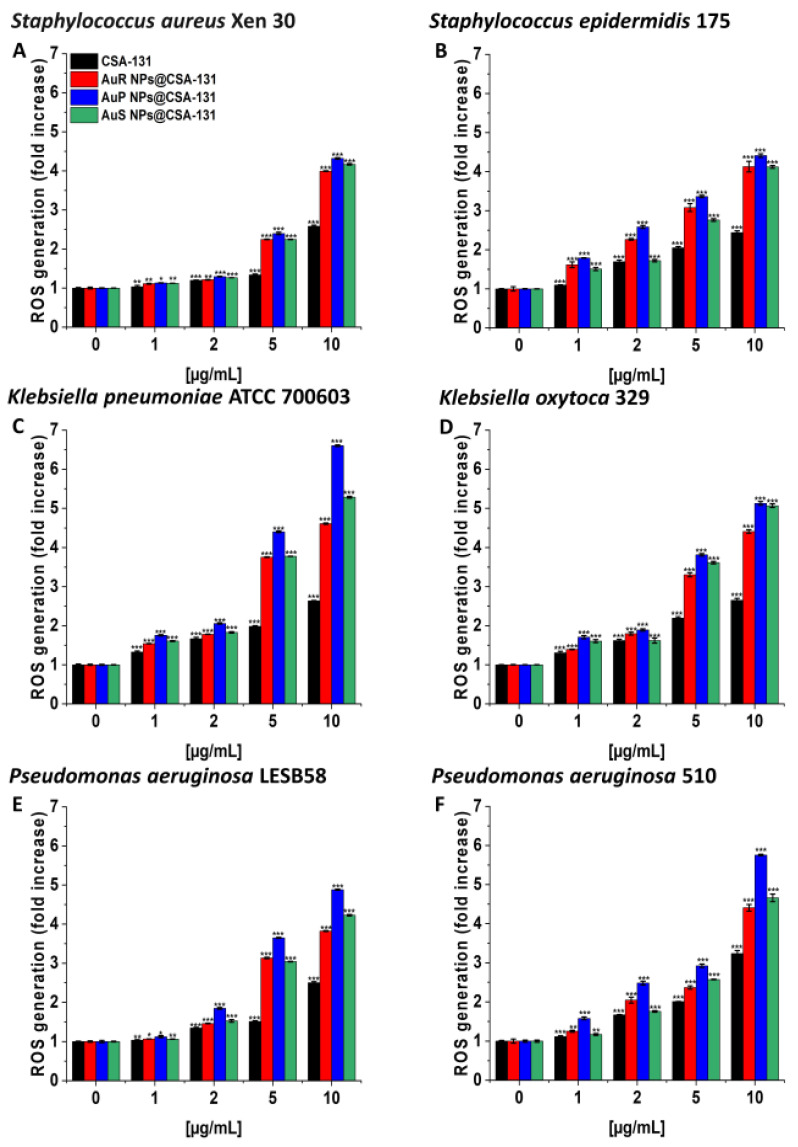
Induction of reactive oxygen species (ROS) generation by *Staphylococcus aureus* Xen 30 (**A**), *Staphylococcus epidermidis* 175 (**B**), *Klebsiella pneumoniae* ATCC 700603 (**C**), *Klebsiella oxytoca* 329 (**D**), *Pseudomonas aeruginosa* LESB58 (**E**), and *Pseudomonas aeruginosa* 510 (**F**) was evaluated by DFCH-DA (2′,7′-dichlorofluorescin diacetate) fluorimetric assay. Formation of ROS by microorganisms subjected to AuR NP@CSA-131, AuP NP@CSA-131, AuS NP@CSA-131, and CSA-131 ranging from 1 to 10 µg/mL was presented. Results show the mean ± SD, n = 3; * indicates statistical significance at *p* ≤ 0.05, ** ≤0.01, and *** ≤0.001.

As presented in Figure 3A–F, treatment with the nanosystems resulted in the generation of ROS among *S. aureus* Xen 30*, S. epidermidis* 175*, K. pneumoniae* ATCC 700603*, K. oxytoca* 329*, P. aeruginosa* LESB58, and *P. aeruginosa* 510 strains in a concentration-dependent manner. This effect was pronounced for all nanosystems; a concentration of 10 µg/mL caused a 3.8- to 6.6-fold increase of ROS, compared to the unstimulated control. In contrast, at the same concentration of CSA-131, only a two- or threefold increase of ROS was observed in relation to the control (Figure 3, panel C).

**Figure 4:**
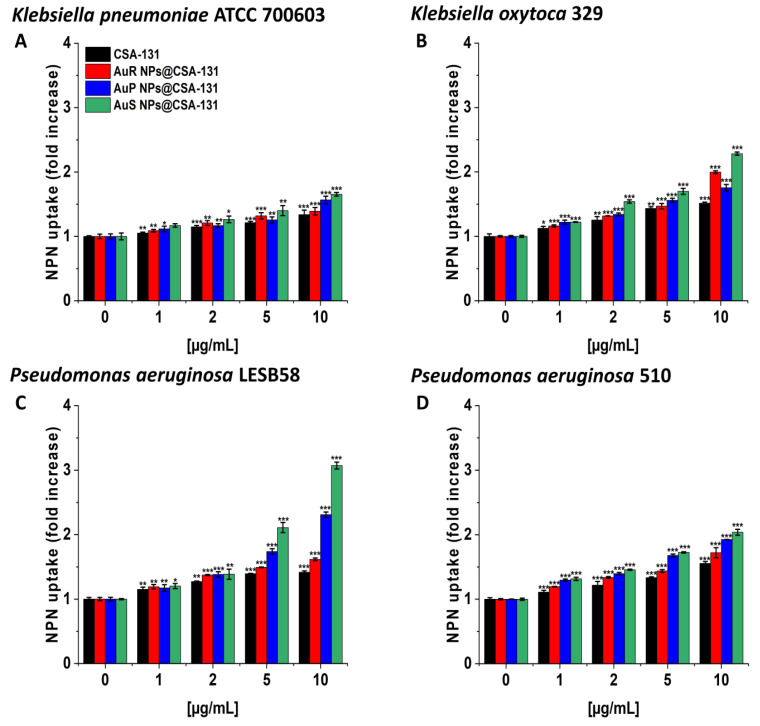
The increase of the outer membrane permeability of *Klebsiella pneumoniae* ATCC 700603 (**A**), *Klebsiella oxytoca* 329 (**B**), *Pseudomonas aeruginosa* LESB58 (**C**), and *Pseudomonas aeruginosa* 510 (**D**) subjected to the tested nanoparticles. Uptake of NPN by microorganisms upon treatment with AuR NP@CSA-131, AuP NP@CSA-131, AuS NP@CSA-131, and CSA-131 at a concentration of 1–10 µg/mL was investigated using the fluorimetric method. Results show the mean ± SD, n = 3; * indicates statistical significance at *p* ≤ 0.05, ** ≤0.01, and *** ≤0.001.

In the NPN assay, the highest level of outer membrane permeability was observed with AuS NP@CSA-131 (Figure 4A–D). Treatment with AuS NP@CSA-131 at 10 µg/mL resulted in an approximately twofold increase in fluorescence compared with the untreated bacteria, *K. oxytoca* 329 (Figure 4, panel B) and *P. aeruginosa* 510 (Figure 4, panel D), and a little over threefold in the case of *P. aeruginosa* LESB58 (Figure 4, panel C).

**Figure 5:**
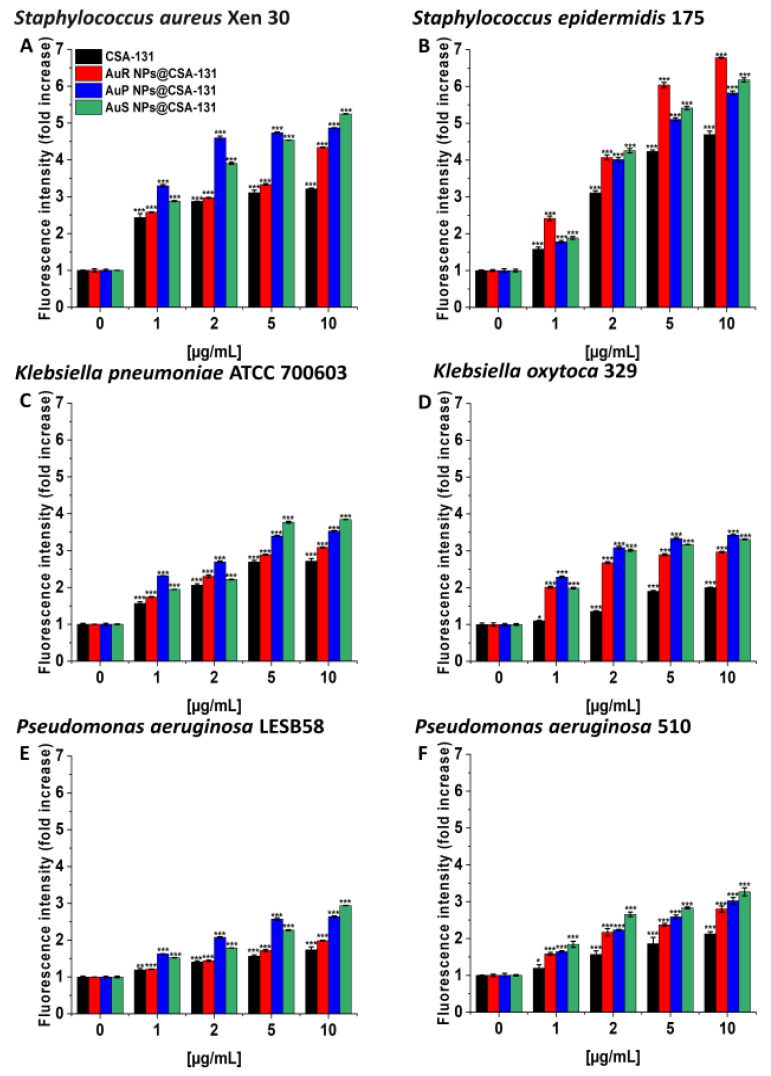
Depolarization of the bacterial membrane of Staphylococcus aureus Xen 30 (**A**), Staphylococcus epidermidis 175 (**B**), Klebsiella pneumoniae ATCC 700603 (**C**), Klebsiella oxytoca 329 (**D**), Pseudomonas aeruginosa LESB58 (**E**), and Pseudomonas aeruginosa 510 (**F**) was assessed using a diSC_(3)_ assay. The evaluation of the degree of cell membrane depolarization in the presence of AuR NP@CSA-131, AuP NP@CSA-131, AuS NP@CSA-131, and CSA-131 ranging from 1 to 10 µg/mL was monitored by the enhancement of fluorescence intensity. Results show the mean ± SD, n = 3; * indicates statistical significance at *p* ≤ 0.05, ** ≤0.01 and *** ≤0.001.

In the diSC_(3)_ assays, AuS NP@CSA-131 showed the highest cell membrane depolarization activity in the majority of cases, notably with *S. aureus* Xen 30 and *S. epidermidis* 175, where 10 µg/mL of the nanosystem caused >5- and >6-fold increases in the fluorescence level compared to the untreated controls (Figure 5A–F).

**Figure 6:**
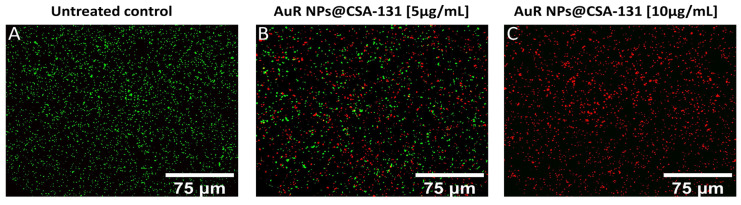
Reduced survival and increase of PI-positive cells upon exposure of the representative strain of *Pseudomonas aeruginosa* 510 to AuR NP@CSA-131 for 1 h was evaluated using a fluorescence microscope. Panel (**A**) demonstrates the untreated control. On the other hand, the viability of the bacteria cells subjected to treatment with AuR NP@CSA-131 at doses of 5 µg/mL (panel **B**) and 10 µg/mL (panel **C**) was compromised. The results from one representative experiment are shown.

The membrane activity of the nanosystems was confirmed by SYTO9/PI-dual fluorescent staining. As presented in Figure 6A–C, increasing the concentrations of AuR NPs@CSA-131 proportionally reduced the number of living *P. aeruginosa* 510 cells (SYTO9-derived green fluorescence), whereas at the same time, the number of dead (PI-derived red fluorescence) cells grew. Significantly, a dose of 5 µg/mL of AuR NPs@CSA-131 (Figure 6, panel B) reduces the survival of *P. aeruginosa* 510 by 50%. However, at a dose of 10 µg/mL, the bactericidal effect was noticed (Figure 6, panel C).

**Figure 7:**
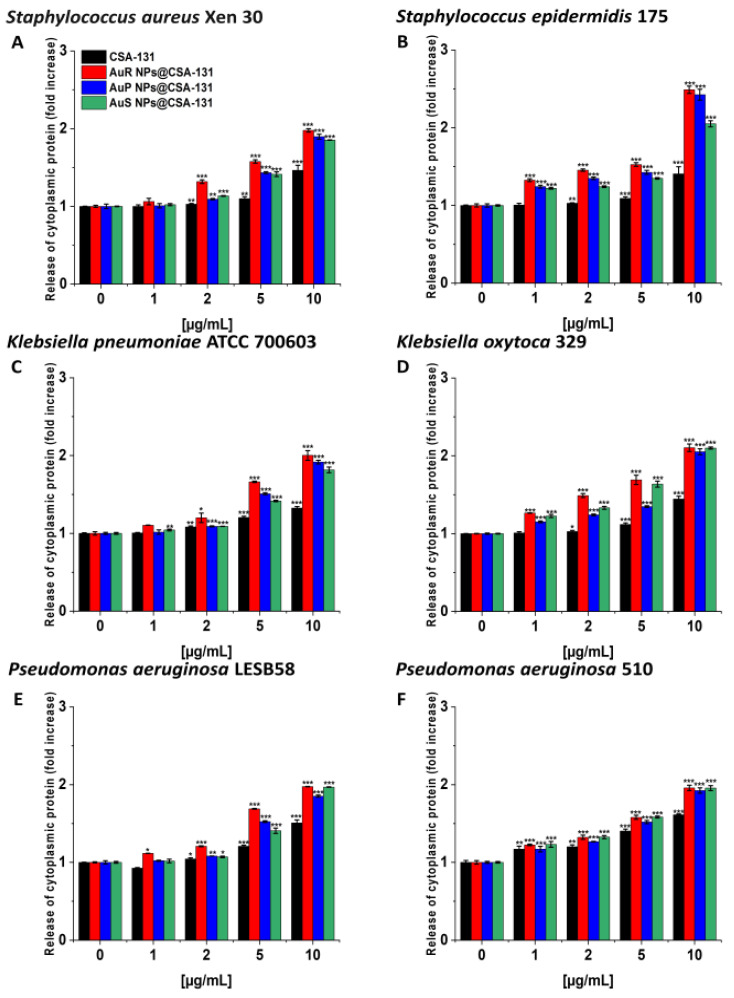
The release of cytoplasmic proteins from Staphylococcus aureus Xen 30 (**A**), Staphylococcus epidermidis 175 (**B**), Klebsiella pneumoniae ATCC 700603 (**C**), Klebsiella oxytoca 329 (**D**), Pseudomonas aeruginosa LESB58 (**E**), and Pseudomonas aeruginosa 510 (**F**) upon treatment with AuR NP@CSA-131, AuP NP@CSA-131, AuS NP@CSA-131, and CSA-131 at concentrations of 1–10 µg/mL was evaluated by Bradford protein assay. Results show the mean ± SD, n = 3; * indicates statistical significance at *p* ≤ 0.05, ** ≤0.01 and *** ≤0.001.

Likewise, the protein leakage from the bacteria treated with the nanosystems was observed in a dose-dependent manner (Figure 7A–F). 

### 3.4. The Nanosystems Exert High Biocompatibility at Bactericidal Doses

**Figure 8:**
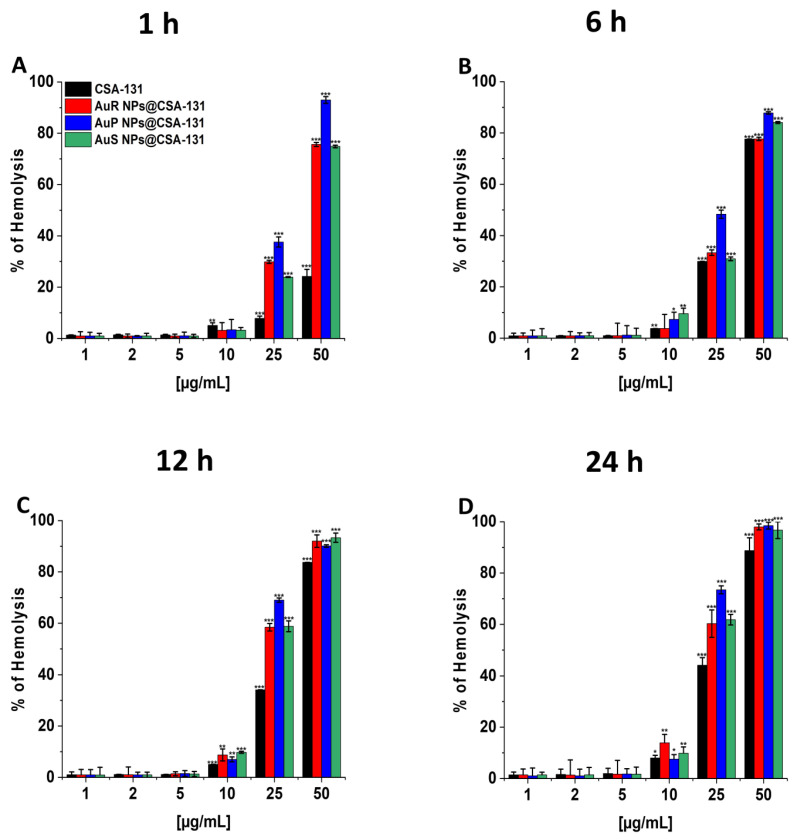
Hemoglobin release from human red blood cells (RBCs) incubated in the presence of AuR NPs@CSA-131, AuP NP@CSA-131, AuS NP@CSA-131, and CSA-131 (**A–D**) at doses of 1–50 µg/mL after 1 h (**A**), 6 h (**B**), 12 h (**C**), and 24 h (**D**). Results show the mean ± SD, n=3; * indicates statistical significance at *p* ≤ 0.05, ** ≤0.01 and *** ≤0.001.

To evaluate the potential toxicity of the nanoparticles investigated, measurement of the hemoglobin released from damaged RBCs, known as a hemolysis assay, was performed at concentrations corresponding to the bactericidal range. The results presented in Figure 8A–D show that all developed nanosystems did not affect RBC membrane permeability at doses from 1 to 10 µg/mL, even if the incubation was extended to 24 h. It is worth pointing out that low hemolysis rates after 1 h of incubation were recorded, i.e., less than 1% at the concentration of 1 µg/mL and a maximum of 3.41% at the dose of 10 µg/mL. Moreover, at 10 µg/mL, only 3.78–9.58%, 7.02–9.78%, and 7.53–13.93% of the erythrocytes were damaged upon 6, 12, and 24 h of exposure to AuR NP@CSA-131, AuP NP@CSA-131, and AuS NP@CSA-131, respectively.

To explore whether gold nanoparticles present in the developed nanosystems might affect the integrity of human erythrocytes, we performed an additional analysis of hemolytic activity of the investigated nanoparticles, with and without the MHDA capping step. Obtained results demonstrated that Au NPs + CTAB and Au NPs + MHDA do not interfere with RBC membrane integrity at doses ranging from 1.46–73.25 pg/mL of Au (the concentration of gold nanoparticles corresponded to each tested dose of the nanosystem), even if the incubation time was extended to 24 h (data not shown).

## 4. Discussion

Despite a compelling number of antibiotics and chemotherapeutics available for medical treatment, bacterial infections, especially those caused by MDR pathogens, still remain a leading factor of morbidity and mortality around the globe [25]. To date, many species of clinically important microorganisms, predominantly *S. aureus, S. epidermidis, K. pneumoniae, K. oxytoca,* or *P. aeruginosa* have developed resistance to antimicrobial agents that significantly obstructs the effective eradication of these bacteria and ultimately intensifies clinical difficulty [50]. The challenging number of MDR strains necessitates the development of novel microbial therapies. The past decade witnessed a substantial upsurge in the global use of nanomaterials as an innovative alternative for combating the constantly growing antibiotic resistance, especially that associated with MRSA, MRSE, MLS_b_, ESBL, AmpC, or CR bacteria [25,51]. Among the many types of nanoparticles, Au NPs have been hallmarked as one of the most promising materials in nanomedicine [27]. It should be highlighted that Au NPs have gained significant attention owning to their unique properties, including an ultra-small size, facile surface functionalization, and minimal toxicity [20,24]. Interestingly, numerous Au NPs have also been used as antibacterial materials conjugated with small molecule antibiotics, antimicrobial peptides, or cationic ligands on their surface [19]. The principal purpose of the utilization of gold nanosystems, and the focus of this investigation, is to aid in the fight of an ongoing crisis of antimicrobial resistance [27]. Although Ag NPs, have an intrinsic antimicrobial ability, they also raise a severe safety danger with regard to practical application due to their cytotoxicity [19]. There is a compelling amount of evidence indicating that Ag NPS can accumulate in the lungs, spleen, kidney, and liver in exposed rats. What is significant is that Ag NPs were reported to occur within the human body, in particular in the brain, due to their ability to cross the blood–brain barrier [21]. Moreover, exposure to nanosilver may trigger apoptosis and gene modulation in the brains of mice [52]. Due to the well documented cytotoxicity of Ag NPs, we decided to investigate gold nanoparticles as a safer and superior choice.

Nevertheless, the experiments on the effects of Au NPs functionalized by ceragenins, in particular CSA-131, on superbugs are incomplete. To the best of our knowledge, the examination of the impact of rod-, peanut-, and star-shaped Au NPs with CSA-13 on MDR bacteria has never been reported. 

Bactericidal activity plays a key role in the therapeutic potential of nanosystems [53]. Hence, we used MIC/MBC values and colony counting assays as common and acceptable methods to assess the bactericidal properties of the studied compounds [54]. AuR NP@CSA-131, AuP NP@CSA-131, and AuS NP@CSA-131 were proven to possess potent antibacterial effects at low concentrations. What is significant is that MICs did not exceed 1.6 µg/mL, and it is worth emphasizing that this value was recorded only for *P. aeruginosa* LESB58 and *P. aeruginosa* 510 in the case of AuS NPs@CSA-131 (Table 6). Additionally, treatment using nanosystems functionalized by CSA-131 reduced MIC values up to 3.2- fold compared to CSA-131 applied alone (Table 6). Furthermore, gold nanoparticles at doses of 2 µg/mL were deemed bactericidal for nearly all of the study strains, which confirms their killing properties (Figure 2A–F). It is appropriate to underline that no considerable differences in bactericidal efficiency of tested nanosystems were observed between MRSA, MRSE, MLS_b_, ESBL, AmpC, or carbapenem-resistant strains (Figure 2A–F), which indicates their potent activity regardless of bacterial species or mechanism of resistance. This may be potentially related to the fact that our nanosystems have a positive surface charge, which facilitates interaction with the negatively charged surface of the bacteria [55]. The positive charge of Au NPs@CSA-131 results from both (1) the nanoparticles’ preparation, which involved the use of CTAB; and (2) the positive charge of CSA-131 ceragenin [56]. On the other hand, bacteria exhibit an overall negative charge. In relation to Gram-positive bacteria, the phosphoryl groups located in teichoic acid and lipoteichoic acid play a pivotal role in the generation of the negative charge. In turn, with regard to Gram-negative bacteria, it is a consequence of the ionization of the phosphoryl and 2-keto-3-deoxyoctonate carboxylate groups occuring in the LPS (lipopolysaccharide). Such surface charge-governed interactions would be favorable in this aspect of the treatment of drug-resistant pathogens [55].

To date, gold has been the subject of attention as an attractive prospective material for combating bacterial infections. According to the literature, Au NPs coated with aminoglycosides have been recognized as efficient antibacterial agents against bacteria such as *S. aureus*, *Micrococcus luteus*, *E. coli,* and *P. aeruginosa* [53]. In addition, a synergistic effect of functionalized Au NPs and fluoroquinolone in the treatment of infections due to MDR *E. coli* was detected [31]. What is more, Shaker et al. demonstrated that carbapenem conjugated with Au NPs exerted superior antibacterial activity against MDR including *K. pneumoniae*, *P. mirabilis,* or *A. baumannii* compared to free carbapenems at the same concentrations. Furthermore, in the above experiment, imipenem-loaded gold nanoparticles showed a fourfold decrease in MIC values, whilst the meropenem-loaded nanoparticles gave a threefold decrease [53,57]. Likewise, Brown et al. observed that ampicillin bound to the surface of Au NPs abolished the bacterial resistance among *E. coli K-12* harboring the β-lactamase gene, as well as *P. aeruginosa* or MRSA strains [43,58]. According to the literature mentioned, it is well documented that Au NPs may potentiate the antimicrobial effects of numerous compounds. However, the data recently published also point out the possibility of the successful application of nanosystems as nanocarriers for CSAs. Our previous study revealed that the immobilization of CSA-13 on magnetic nanoparticles (MNP-CSA-13) considerably enhanced the antimicrobial activity against MDR *P. aeruginosa* [59]. It is also noteworthy that CSA-13 immobilized on MNP not only significantly improved its bactericidal and anti-biofilm activities under PBS-based conditions, but also intensified the antimicrobial activity in the presence of bodily fluids (such as urine, saliva, plasma, pus, ascites, cerebrospinal fluid, bronchoalveolar lavage, and cystic fibrosis sputum) [51,59]. The therapeutic usefulness of MNPs is further highlighted by the data, indicating that the combination of CSA-13 and CSA-131 with MNPs increased their bactericidal effects and the ability to prevent the biofilm formation by *S. aureus* Xen 30 and *P. aeruginosa* Xen 5 [51,54]. Likewise, Durnaś et al. demonstrated that the immobilization of CSA-13 and CSA-131 onto the surface of MNPs disclosed a comparable or stronger bactericidal activity against anaerobic bacteria, including the representative species of *Bacteroides* spp. and *Prevotella* spp., as well as *Clostrium perfringens* and *Peptostreptococcus* spp. Moreover, the enhanced capacity of these compounds to prevent biofilm formation by *Bacteroides fragilis* and *Cutibacterium acnes* was demonstrated in the aforementioned experiment [51,60]. A similar phenomenon was detected in our experimental settings. Importantly, more potent activity of the tested nanosystem when compared to ceragenin CSA-131 results from the local increase of ceragenin density and its improved cellular uptake, than from the antimicrobial activity of nanogold itself. Although our previous research demonstrated that variably shaped gold nanoparticles might exert antimicrobial [61] and anti-cancer effects [62], it should be kept in mind that for achieving such killing activity, higher doses of nanoparticles are required than those present in the developed nanosystems. 

Other reports have demonstrated that shape may also play a role in the antibacterial activity of gold nanoparticles [53]. Hameed et al., concluded that gold nanocubes (Au NCs) showed the most significant bactericidal property, even at lower concentrations, against *E. coli*, *P. aeruginosa,* and *S. aureus,* followed by Au NSps (nanospheres) and Au NSts (nanostars) [63]. In contrast to the literature, the results of the present study showed no significant effect on the viability of treated bacteria with respect to the shape of the nanosystems. Crucially, rod-, peanut-, and star- shaped Au NP@CSA-131 displayed comparable bactericidal properties against *S. aureus* Xen 30*, S. epidermidis* 175*, K. pneumoniae* ATCC 700603*, K. oxytoca* 329*, P. aeruginosa* LESB58, and *P. aeruginosa* 510. Taking into account the outcomes obtained, it may be concluded that all of the studied nanosystems presented the potential for application as novel antibacterial compounds in the treatment of bacterial infections, predominantly those triggered by MDR strains. Importantly, their more potent activity is likely independent of residual CTAB, used for the synthesis of nanoparticles as a shape-controlling agent. To validate the accuracy of this statement, we estimated the CTAB amount in the nanosystem solutions using FT-Raman spectroscopy. For this purpose, spectra of pure CTAB nanoparticles before and after washing were measured, and MagicPlotSoftware was used to calculate the value of the sum of peak area characteristics for CTAB. Finally, the percentage of CTAB in the nanoparticle solutions was determined in comparison with the pure CTAB solution and nanoparticle solutions before rising. The calculated percentage of CTAB demonstrated that in Au NPs solutions before rising, CTAB was approximately 40%. Moreover, the percentage of CTAB in the nanoparticle solutions after rising was ~6%. It should be noted that our nanosystems consist of 2 mg/mL ceragenin CSA-131 and 2.93 ng/mL of non-spherical gold nanoparticles. Given the above, it can be estimated that the amount of CTAB present on the nanoparticles’ surface used for CSA-131 immobilization, even at the highest tested doses of nanosystem (100 ug/mL), does not exceed 60 pg/mL. According to our experience, such amounts of CTAB do not exert any bactericidal effects, since the MIC values for CTAB recorded in our lab (using a spectrum of Gram-positive, Gram-negative bacteria, and *Candida* fungi) were mostly > 32 µg/mL (data not shown). This approach allows us to conclude that this minor amount of CTAB in solutions should not affect the bactericidal activity.

The potential of NPs in the field of medicine has resulted in the interest of many scientists in exploring the mechanisms of NP actions. Hence, one goal of our study was to investigate the mode of action of synthesized AuR NP@CSA-131, AuP NP@CSA-131, and AuS NP@CSA-131 [26]. It has been proposed that the antibacterial effects of NPs are associated with mechanisms involving oxidative stress, the release of metal ions, and non-oxidative processes [21,26]. However, compelling evidence suggests that the vital mechanism of NPs is connected to ROS-induced oxidative stress. Under normal circumstances, a balance is maintained between the generation and clearance of ROS in bacterial cells. Nevertheless, if ROS production occurs in an excessive amounts, the intracellular redox state is altered, promoting oxidation [21]. What is more, reports have indicated that oxidative stress is a predominant contributor to cell membrane permeability changes. Notably, the generation of ROS may affect the loss of membrane integrity and attack proteins and enzymes, which play a crucial role in the cell morphology, ensuring the normal physiological processes of the bacterial cells. Moreover, ROS may cause elevated expressions of oxidative proteins that accelerate apoptosis and ultimately lead to the death of the cell [26]. In order to confirm this assumption with regard to the study, MDR bacteria producing methicillin-resistant, MLS_b_, ESBL, AmpC, or carbapenem-resistant were utilized and the following methods were performed: ROS generation assessment (Figure 3A–F), NPN uptake (Figure 4A–D), diSC_(3)_ assay (Figure 5A–F), and protein leakage assay (Figure 7A–F). The results obtained strongly suggest that the mechanisms by which AuR NP@CSA-131, AuP NP@CSA-131, and AuS NP@CSA-131 exert potent antimicrobial effects against the studied bacteria involved in the production of ROS, resulting in the destruction of bacterial membranes and the leakage of the intracellular protein content, which consequently led to bacterial death. Our data are in agreement with the previous study reported by Piktel et al. associated with rod-shaped gold nanoparticles and their fungicidal mechanism of activity [61]. Likewise, Mohamed et al. demonstrated that the bactericidal activity of gold nanoparticles against *Corynebacterium pseudotuberculosis* was attributed to ROS production [22]. Similar conclusions were drawn by Zheng et al., who showed that gold nanoclusters (Au NCs) effectively killed MDR superbugs belonging to the ESKAPE group [MDR *Acinetobacter baumannii*, MDR *Pseudomonas aeruginosa*, MDR *Klebsiella pneumoniae*, MDR *Enterobacter* spp., methicillin-resistant *Staphylococcus aureus* (MRSA) and vancomycin-resistant *Enterococcus faecium* (VRE)] by the induction of overwhelming intracellular ROS generation [19]. Moreover, findings obtained by Xie et al. also emphasized the imbalance in the redox status among MRSA bacteria upon gold nanocluster treatment [20]. It is worth emphasizing that these observations primarily related to the effects of CSA on the mechanism of nanoparticle actions, and are supported by our previous publications showing that MNP-CSA-13 adhered to bacteria and then damaged the cell membrane with leakage of the intracellular contents [59]. The recent data also showed that the incubation of fungal cells with MNPs conjugated with CSA-13 interrupted the oxidation–reduction balance, which resulted in the superior generation of ROS followed by pore formation within the cell membranes and finally the disruption of the membranes [64].

To create a satisfactory gold nanosystem for medical applications, a crucial aspect that must be considered is toxicity. Therefore, the hemolytic activity of AuR NP@CSA-131, AuP NP@CSA-131, and AuS NP@CSA-131 was determined at doses corresponding to MICs and to the bactericidal range (Figure 8A–D). It was also revealed that gold NPs seemed to be comparatively safer than other metallic NPs due to the inert and nontoxic nature of gold [65]. Moreover, previous studies have suggested that spherical Au NPs are generally more toxic and ingested more efficiently than rod-shaped nanoparticles [2]. The investigations presented here demonstrated only negligible damage to erythrocyte membranes at doses demonstrating bactericidal effects. Importantly, there was no discernible toxic effects correlated with the shape of the nanosystems (Figure 8A–D). Due to minimal hemolytic effects, AuR NP@CSA-131, AuP NP@CSA-131, and AuS NP@CSA-131 appear safe for human cells, which further confirms our presumptions regarding the potential utility of synthesized nanosystems in medicine. Similar conclusions might be drawn based on our previous data showing that gold nanorods induced no considerable toxicity. Exposure of RBCs to AuR NPs at doses from 0.125 to 2.5 ng/mL resulted in hemolysis no higher than 1% [61].

On the other hand, to address the possible toxicity of CSAs used alone, our previous data underlined that CSA-131 at concentrations ranging from 1–10 µg/mL caused no significant hemolysis, even after 12 h of incubation [35]. Moreover, the recent data also demonstrated that MNP-CSA-13 at concentrations of 1–100 µg/mL had no effect on the hemoglobin released from RBC [59]. It is worth noting that ceragenins with core–shell magnetic NPs significantly increased the biocompatibility of such combinations, which proves that the chemical adsorption of drugs onto nanoparticle surfaces may decrease hemolysis [51,59]. 

It is worth underlining that the results presented herein provide grounds for further studies on the development of gold nanosystems functionalized by CSA-131 with potent bactericidal activity to overwhelm the microorganism resistance from the perspective of the treatment of infections [66]. 

Due to the unique aforementioned properties of synthesized nanosystems, their potential application includes the coating of devices such as catheters. Crucially, catheters are incredibly prone to bacterial colonization, leading to a greater risk of catheter-associated UTI (CAUTI), which account for 40% of all nosocomial infections worldwide [23,67]. AuR NP@CSA-131, AuP NP@CSA-131, and AuS NP@CSA-131 can all be used as antibacterial materials to retard or even inhibit the growth of bacteria on the catheter, notably those producing methicillin-resistant MLS_b_, ESBL, AmpC, or CR, respectively. Moreover, our nanosystems may be of considerable interest in wound dressings. It should be underlined that wound infections (e.g., diabetic foot ulcers) are mainly accompanied by tested species of bacteria, with special regard to their mechanisms of resistance, thereby increasing the risk of amputation [23,68]. The application of wound dressings coated by AuR NP@CSA-131, AuP NP@CSA-131, or AuS NP@CSA-131 may inhibit the growth of bacteria and potentate the rate of wound healing [23]. However, it is necessary to point out that results obtained are preliminary, and many aspects surrounding the utilization of the tested nanosystems as materials coatings need further investigations.

Collectively, beyond a shadow of a doubt, our observations may contribute to the development of novel bactericidal agents.

## 5. Conclusions

**Figure 9:**
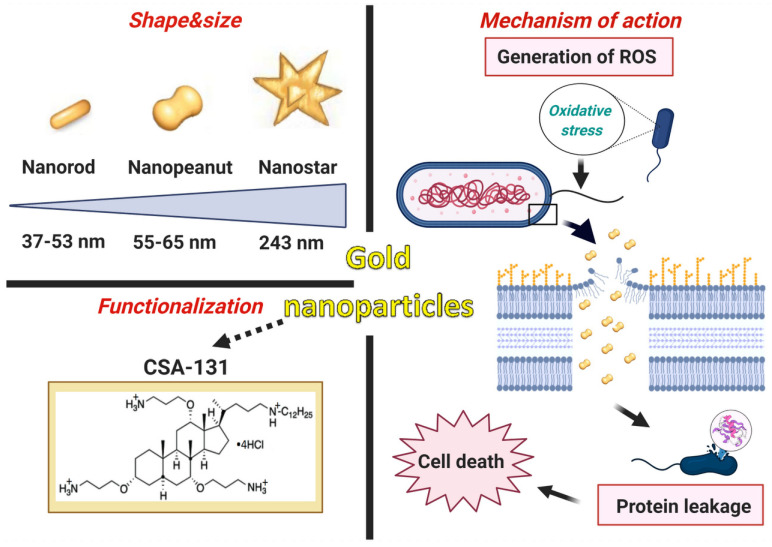
Characteristics of gold nanoparticles functionalized by CSA-131. Proposed antimicrobial mechanism of the developed nanosystems which comprises the generation of ROS (reactive oxygen species) associated with the destruction of the bacterial membranes as well as the leakage of the intracellular content and consequently cell death. The figure was prepared using BioRender.

In summary, the innovation of the presented research consists of the development of newer and superior nanosystems composed of rod-, peanut-, and star-shaped gold nanosystems functionalized by CSA-131 (Figure 9) that potentiate bactericidal activity when compared to the antimicrobial activity of CSA-131 in a free form. This is the first report evaluating the activity of AuR NP@CSA-131, AuP NP@CSA-131, and AuS NP@CSA-131 against bacteria strains producing methicillin-resistant MLS_b_, ESBL, AmpC, or those characterized by carbapenem-insensitivity. It is important to note that the number of broad-spectrum drugs with therapeutic efficacy against both Gram-positive and Gram-negative bacteria harboring various genes of resistance is very negligible, which underlines the importance of our investigations. Notably, all of the synthesized nanosystems exert significantly enhanced bactericidal effects against the studied strains compared to CSA-131, irrespective of the identified mechanism of drug resistance. The studies presented herein demonstrate that antibacterial mechanisms of AuR NP@CSA-131, AuP NP@CSA-131, and AuS NP@CSA-131 cause the generation of reactive oxygen species, which are related to the destruction of bacterial membranes and the leakage of intracellular content (Figure 9). The potential usefulness of the study of gold nanosystems in medicine is further promoted by the satisfactory results of hemocompatibility.

## Figures and Tables

**Table 1:** The characteristics of the examined bacterial strains.

Strain	Mechanism of Resistance	Type of Strain/Source
*S. aureus* Xen 30	- MRSA, - constitutive MLS_b_	- Reference strain/purchased from Caliper Life Sciences (Hopkinton, MA)
*S. epidermidis* 175	- MRSE, - constitutive MLS_b_	- Clinical strain/isolated from tracheobronchial secretions
*K. pneumoniae* ATCC 700603	- ESBL caused by SHV-18 - impermeability to cephamycin as a mechanism associated with the alteration of porins	- Reference strain/purchased from American Type Culture Collection (ATCC, USA)
*K. oxytoca* 329	- ESBL - acquired plasmid-mediated cephalosporinase AmpC	- Clinical strain/collected from the urine
*P. aeruginosa* LESB58	- highly resistant to antibiotics with production of chromosomally encoded inducible AmpC-β-lactamase	- Hypervirulent and an epidemic cystic fibrosis strain isolated from sputum
*P. aeruginosa* 510	- ESBL related to the active efflux pump - alteration of the outer membrane permeability resulting in OprD2 deficiency and finally resistance to carbapenems	- Clinical strain/collected from tracheobronchial secretions

Abbreviations: MRSA: methicillin-resistant *S. aureus*; MLS_b_: resistant to macrolides, lincosamides, and streptogramin group B; MRSE: methicillin-resistant *S. epidermidis;* ESBL: extended spectrum β-lactamase; AmpC: cephalosporinase.

**Table 2:** Antibiotic susceptibility of *Staphylococcus aureus* Xen 30 and *Staphylococcus epidermidis* 175.

Antibiotic/Chemotherapeutic	*Staphylococcus aureus* Xen 30	*Staphylococcus epidermidis* 175
	Interpretation	Interpretation
Screening test with cefoxitin	Positive	Positive
Oxacillin	R	R
Erythromycin	R	R
Clindamycin	R	R
Amikacin	R	R
Gentamicin	R	R
Ciprofloxacin	R	R
Levofloxacin	R	R
Linezolid	S	S
Daptomycin	S	S
Teicoplanin	S	S
Vancomycin	S	S

Abbreviations: R: resistance, S: susceptibility.

**Table 3:** Antibiotic susceptibility of *Klebsiella pneumoniae* ATCC 700603 and *Klebsiella oxytoca* 329.

Antibiotic/Chemotherapeutic	*Klebsiella pneumoniae* ATCC 700603	*Klebsiella oxytoca* 329
	Interpretation	Interpretation
Ampicillin	R	R
Amoxicillin/Clavulanic acid	I *	R
Piperacillin/Tazobactam	I *	R
Cefuroxime	R	R
Cefuroxime axetil	R	R
Cefotaxime	R	R
Ceftazidime	R	R
Ertapenem	S	S
Meropenem	S	S
Amikacin	S	S
Gentamicin	R	R
Ciprofloxacin	S	R
Norfloxacin	S	R

Abbreviations: R: resistance, S: susceptibility. * Despite the sensitivity *in vitro*, there is a risk of therapy failure.

**Table 4:** Antibiotic susceptibility of *Pseudomonas aeruginosa* LESB58 and *Pseudomonas aeruginosa* 510.

Antibiotic/Chemotherapeutic	*Pseudomonas aeruginosa* LESB58	*Pseudomonas aeruginosa* 510
	Interpretation	Interpretation
Piperacillin	R	R
Piperacillin/Tazobactam	R	R
Ticarcillin/Clavulanic acid	R	R
Ceftazidime	R	R
Cefepime	R	R
Imipenem	S	R
Meropenem	S	R
Amikacin	R	R
Gentamicin	R	R
Tobramycin	R	R
Ciprofloxacin	R	R
Levofloxacin	R	R
Colistin	S	S

Abbreviations: R: resistance, S: susceptibility.

**Table 5:** Assays used for the analysis of the bacterial response to the nanosystems.

Assay	Indicator Reagent	Final Bacterial Inoculum	Final Concentration of the CSA-131	Test Conditions	Results Recording
ROS generation	2′,7′-dichlorofluorescin diacetate (DCFH-DA, Sigma-Aldrich, USA)	OD_600_ = 0.1 in PBS	1–10 µg/mL	60 min incubation at 37 °C with 20 µM DCFH-DA in PBS (in 96-well black plates)	fluorescence emission at wavelengths of 488/535 nm
Outer membrane permeabilization *	1-N-phenylnapthylamine (NPN, Sigma-Aldrich, USA)	OD_600_ = 0.1 in PBS	1–10 µg/mL	5 min incubation at 37 °C with 0.5 mM NPN	fluorescence intensity λex = 348 nm/λem = 408 nm
diSC_(3)_	3,3′-dipropylthiadicarbocyanine iodide (diSC_(3)_, Sigma Aldrich, USA)	OD_600_–0.05	1–10 µg/mL	60 min incubation at room temperature with 0.4 µM diSC_(3)_, followed by 5 min incubation with 100 mM KCl	fluorescence emission at wavelengths of 622/670 nm
SYTO9/PI-dual staining **	LIVE/DEAD BacLight Bacterial Viability Kit (CA, USA)	OD_600_–0.5	5 µg/mL and 10 µg/mL	60 min incubation at 37 °C in PBS, followed by staining using SYTO9 dye and propidium iodide (PI) for 15 min	fluorescence microscopy (Zeiss AxioObserver.A1 Fluorescence Version Inverted Optical Microscope, JPK Instruments, German).
Protein leakage	Coomassie Brilliant Blue G-250 (Bradford reagent, Sigma-Aldrich, USA)	OD_600_–0.1	1–10 µg/mL	60 min incubation at 37 °C, followed by 10 min centrifugation (5000 rpm at 4 °C) and incubation of supernatant with Bradford reagent (1:1 ratio) for 10 min in dark	absorbance level at 595 nm

* Test performed only for the Gram-negative bacteria, i.e., *K. pneumoniae* ATCC 700603, *K. oxytoca* 329, *P. aeruginosa* LESB58, and *P. aeruginosa* 510. ** test performed only with *P. aeruginosa* 510.

**Table 6:** MIC and MBCs values of CSA-131, AuR NP@CSA-131, AuP NPs@CSA-131, and AuS NP@CSA-131 against the studied strains of bacteria.

Ceragenins/Nanosystems/ Microorganisms	*S. aureus* Xen 30 MIC/MBC (µg/mL)	*S. epidermidis* 175 MIC/MBC (µg/mL)	*K. pneumoniae* ATCC 700603 MIC/MBC (µg/mL)	*K. oxytoca* 329 MIC/MBC (µg/mL)	*P. aeruginosa* LESB58 MIC/MBC (µg/mL)	*P. aeruginosa* 510 MIC/MBC (µg/mL)
CSA-131	0.5/1	0.5/1	2/2	1/4	4/8	2/8
AuR NPs@CSA-131	0.2/0.4	0.2/0.2	0.8/1.6	0.8/3.2	0.8/1.6	1.6/3.2
AuP NPs@CSA-131	0.4/1.6	0.4/0.8	0.8/3.2	0.8/6.4	0.8/0.8	1.6/3.2
AuS NPs@CSA-131	0.2/0.8	0.2/0.4	0.8/1.6	0.4/0.8	1.6/1.6	1.6/3.2

## Data Availability

The data that support the findings of this study are available from the corresponding author upon reasonable request.
