# Correction: Bucki et al. Bactericidal Properties of Rod-, Peanut-, and Star-Shaped Gold Nanoparticles Coated with Ceragenin CSA-131 against Multidrug-Resistant Bacterial Strains. *Pharmaceutics* 2021, *13*, 425

**DOI:** 10.3390/pharmaceutics16111354

**Published:** 2024-10-24

**Authors:** Sylwia Joanna Chmielewska, Karol Skłodowski, Joanna Depciuch, Piotr Deptuła, Ewelina Piktel, Krzysztof Fiedoruk, Patrycja Kot, Paulina Paprocka, Kamila Fortunka, Tomasz Wollny, Przemysław Wolak, Magdalena Parlinska-Wojtan, Paul B. Savage, Robert Bucki

**Affiliations:** 1 Department of Medical Microbiology and Nanobiomedical Engineering, Medical University of Bialystok, 15-222 Bialystok, Poland; sylwia.chmielewska@umb.edu.pl (S.J.C.); karol.sklodowsky@gmail.com (K.S.); piotr.deptula@umb.edu.pl (P.D.); ewelina.piktel@wp.pl (E.P.); krzysztof.fiedoruk@umb.edu.pl (K.F.); 2 Institute of Nuclear Physics, Polish Academy of Sciences, 31-342 Krakow, Poland; joannadepciuch@gmail.com (J.D.); magdalena.parlinska@ifj.edu.pl (M.P.-W.); 3 Department of Microbiology and Immunology, Institute of Medical Sciences, Collegium Medicum, Jan Kochanowski University in Kielce, 25-365 Kielce, Poland; patrycja.kot52@gmail.com (P.K.); paulina.paprocka@ujk.edu.pl (P.P.); kamilafortunka@gmail.com (K.F.); przemyslaw.wolak@ujk.edu.pl (P.W.); 4 Holy Cross Cancer Center, Kielce, 25-734 Kielce, Poland; tomwollny@gmail.com; 5 Department of Chemistry and Biochemistry, Brigham Young University, Provo, UT 84602, USA; pbsavage@chem.byu.edu

In the original publication [1], the reference [2] in Figure 1b showing a STEM photograph of AuP NPs, was not cited. The citation has now been inserted in Figure 1 caption, and should read: 

**Figure 1.** Overview STEM image of the obtained AuR NPs (**a**,**a1**), AuP NPs (**b** adapted from: [44], **b1**), and AuS NPs (**c**,**c1**); Selected Area Diffraction (SEAD) patterns of the AuR NPs (**a2**), AuP NPs (**b2**), AuS NPs (**c2**); size distribution of the AuR NPs (**a3**), AuP NPs (**b3**), AuS NPs (**c3**); unenhanced Raman spectra of the MHDA (black spectra)-functionalized nanoparticles by MHDA (blue spectra) and immobilized CSA-131 in the NP surface (red spectra) for the AuR NPs (**a4**), AuP NPs (**b4**), and AuS NPs (**c4**).

With this correction, the order of some references has been adjusted accordingly. The authors state that the scientific conclusions are unaffected. This correction was approved by the Academic Editor. The original publication has also been updated.
